# Supplementary material for: Cortical reliability amid noise and chaos
Source: Nat Commun. 2019 Aug 22;10:3792. doi: 10.1038/s41467-019-11633-8 (PMC6706377; doi:10.1038/s41467-019-11633-8)
Supplement: Supplementary file 1 — Supplementary Information [file 41467_2019_11633_MOESM1_ESM.pdf]

Supplemental Information:
Cortical reliability amid noise and chaos

Max Nolte[1,*], Michael W. Reimann[1], James G. King[1],
Henry Markram[1,2], and Eilif B. Muller[1,*]

Supplementary Note 1

Divergence is nearly saturated at the scale of the microcircuit

It is possible that the amount of internally generated variability in terms of divergence depends not just on the dynamical state of the model circuit but also on its size. We have previously shown that increasing the size of the model beyond the size described above does not alter the observed dynamical states[1]. At this size, dendritic trees and thus the afferent connections of neurons in the lateral center of the microcircuit are fully located within the microcircuit. However, a large fraction of their recurrent connections with neurons in the surrounding tissue are with neurons beyond the periphery of the microcircuit. Since these neurons were not included in the simulations, large portions of synaptic input to peripheral neurons were missing. To quantify the effect of this additional input on variability in the microcircuit, we surrounded the original microcircuit with six additional microcircuits, simulating a much larger mesocircuit, providing missing synaptic input to the neurons at the periphery of the microcircuit (Fig. 2b1, blue and grey). Connectivity in this mesocircuit was homogeneous, both within and between the individual microcircuits.

When we compared the divergence of membrane potentials between micro- and mesocircuit simulations, we found that membrane potentials diverged slightly faster in the mesocircuit, although the time courses of divergence followed similar trends (Fig. 2b2). The mean difference in $r_{\mathrm{V}}(t)$ was always below 0.06, and the steady state difference below 0.03. Considering the difference at 10-20 ms (which we found to be a good predictor of the relative order of differences at any time), we found this difference to increase towards the periphery of the microcircuit (Fig. 2b3). This suggests that additional direct synaptic input onto a neuron increases variability, but has only a weak effect on indirectly connected neurons. Thus, at the scale of the microcircuit, the amount of internally generated variability is nearly saturated, albeit underestimated for neurons at the periphery.

Highly connected neurons diverge faster

Next, we explicitly quantified how the time course of divergence depends on the amount of the synaptic input. To this end, we examined the relationship between the similarity $s_{\mathrm{r}}(t)$ of a given neuron and the number of connections it receives from within the microcircuit (*in-degree*). Once more, we found that the time course of divergence was faster, the more synaptic inputs a neuron received, as summarized by $s_{\mathrm{r}}(t)$ at 10-20 ms (Fig. 2c). Thus, neurons which are more strongly coupled to the local population[2] diverge more quickly. Additionally, we found that for highly connected neurons, divergence increased with their ratio of excitatory vs. inhibitory inputs (Supplementary Fig. 3b). Repetition of the analysis using $\mathrm{RMSD}_{\mathrm{V}}(t)$ instead of $r_{\mathrm{V}}(t)$ gave qualitatively similar results.

[1]Blue Brain Project, École Polytechnique Fédérale de Lausanne, 1202 Geneva, Switzerland; [2]Laboratory of Neural Microcircuitry, Brain Mind Institute, École Polytechnique Fédérale de Lausanne, 1015 Lausanne, Switzerland; [*]Correspondence: *max.nolte@epfl.ch*, *eilif.mueller@epfl.ch*

Supplementary Note 2

Predicting the impact of missing noise sources

We studied how the magnitude of a generic white noise depolarizing current affects the time course of divergence. Previously, the variance σ_s^2 had been set to 0.001% of the firing threshold for each neuron—a level far lower than other sources of noise. When we increased the variance to values from 0.01% up to 10%, and disabled all other noise sources, we observed that increasing variance led to more rapidly diverging network dynamics (Supplementary Fig. 8a). However, when other noise sources were also enabled, the noisy current injection only affected network dynamics beyond a certain threshold (Supplementary Fig. 8b).

To characterize this threshold, we determined the magnitude of white noise required to cause a noticeable change in the network divergence rate. To this end, we used a decoupled replay paradigm with only noisy current injection (as above), for various levels of σ_s^2 (Fig. 4d1). As above, we quantified the somatic voltage fluctuations due to this noise source, denoted by $\mathrm{RMSD}^{\mathrm{d_x}}_{\infty,\mathrm{dec}}$ (d_x: only white noise, with magnitude x). In the corresponding network simulations, the rate of divergence was strongly dependent on $\mathrm{RMSD}^{\mathrm{d_x}}_{\infty,\mathrm{dec}}$, with larger values leading to faster divergence (Fig. 4d2, dashed line). In contrast, when *all* noise sources were enabled (Fig. 4d2, solid line), there was only a meaningful influence of the noise injection when it was beyond a threshold in the range 0.1%–0.5%. At this threshold, $\mathrm{RMSD}^{\mathrm{d}}_{\infty,\mathrm{dec}}$ was just above 1 mV, approximately half of the value for synaptic noise sources ($\mathrm{RMSD}^{\mathrm{ab}}_{\infty,\mathrm{dec}}$, Fig. 4d2, vertical purple line "ab"). When σ_s^2 is increased even more, the curves for $s_{\mathrm{r},10-20\mathrm{ms}}$ with noisy current alone and with all noise sources eventually converge. Thus, when $\mathrm{RMSD}^{\mathrm{d}}_{\infty,\mathrm{dec}}$ was larger than $\mathrm{RMSD}^{\mathrm{ab}}_{\infty,\mathrm{dec}}$ the noisy current injection dominated other noise sources. This suggests that the strongest source of cellular noise dominates over other sources, unless they are of a comparable magnitude. Taken together, under biological conditions, we predict that synaptic noise is the most important cellular noise source, determining the variability of neuronal responses to presynaptic inputs in vivo. This prediction is consistent with previous findings that cortical neurons respond highly reliably to current injections in vitro, where synaptic noise plays no role[3].

Supplementary Discussion

Quenching of spike-count variability

The predicted mechanism of suppression of chaotic dynamics does not yet have a direct experimental confirmation. However, the effect is related to the often observed quenching of variability—in terms of trial-to-trial spike counts—at the onset of stimuli[4]. In the NMC-model, spike count variance is low both during spontaneous (Fig. 1f) and evoked activity to the same stimulus (Supplementary Fig. 12b1). However, in the intact animal, a neocortical microcircuit is integrated with the rest of the brain and constantly receiving input: around 80% of corticocortical synapses are formed with non-local neurons[1], which are not yet accounted for in the NMC-model. In the behaving brain, most of this external input to the microcircuit will likely contain signals: for example, visual cortex is strongly modulated by movement-related activity[5]. Indeed, when we stimulated the NMC-model with in vivo recordings of thalamic input that was recorded across multiple trials (Supplementary Fig. 12a1-3), instead of perfectly identical input, Poisson-like spike count variances sometimes emerged (Supplementary Fig. 12b2). When we stimulated the NMC-model with variable thalamic input to account for the effect of hidden inputs, Fano factors increased to values observed in rat somatosensory cortex in vivo[6] (Supplementary Fig. 13a1,a3,b1,b2,c2, $t < 0$ ms). When we added reliable input on top of the variable input, spike count variability was quenched (Supplementary Fig. 13a1,a3,b1,b2,c2, $t > 0$ ms), consistent with previous reports[4]. Importantly, repeating one specific input out of the set of variable inputs once again led to a low spike count variance (Supplementary Fig. 13a2,c1). Taken together, these results support the hypothesis that the observed cortical spike count variability in vivo is actually a reliable response to unobserved input, i.e. variable

inputs projecting from diverse locations throughout the brain[7,8]. From this point of view, the observed quenching of variability at stimulus onset[4] reflects the statistical impact of knowledge of the stimulus[9].

Potential effects of missing biological detail

The most important missing detail in the NMC-model is ion-channel noise. Other electrical noise sources such as thermal noise are orders of magnitude smaller[10]. The ion-channel noise in irregular firing neurons in the NMC-model (which is responsible for the irregular initiation of action potentials in vitro[11]) is overshadowed by synaptic noise under in vivo-like conditions (Fig. 4). But how would additional ion-channel noise in axons and dendrites of all neurons impact variability? In dendrites, ion-channel noise is thought to evoke little to no variability in isolated back-propagating action potentials[12]. Thus, mean ion-channel models are likely sufficient for accurate action potential initiation.

Action potentials reliably permeate axonal arbors of neocortical pyramidal neurons without failures[13]. But as action potentials propagate along axons, their timing becomes increasingly variable. Simulations predict that ion-channel noise affects action potential timing in all axons with a diameter below 0.5 μm, with the standard deviation of action potential variability predicted to increase by 0.6 ms per 2 mm in 0.2 μm diameter axons[14]. In the NMC-model, axons have a mean axonal diameter of around 0.3 μm and are modeled deterministically. Therefore, ion-channel noise in longer axons could increase variability of spike timing by up to several milliseconds.

The missing ion-channel noise might push the circuit towards a more variable state. On the other hand, adding missing detail to the synapse models might increase reliability: The reliability of synaptic transmission increases with the number of readily releasable vesicles[15]. Some studies have found univesicular synaptic transmission at cortical synapses[16], while others have estimated there may be as many as ten releasable vesicles per synapse[17].

The current version of the NMC-model assumes one readily releasable vesicle per synapse, and thus potentially underestimates synaptic reliability. To estimate the potential impact of multivesicular release, we repeated the simulation experiments with an increasing number of readily releasable vesicles ($n_{\rm rrp}$) at all synapses (Supplementary Fig. 16). As expected, the time course of divergence slowed with increasing $n_{\rm rrp}$. Nonetheless, for mean $n_{\rm rrp}$ values which reproduce cortical PSP variability data ($n_{\rm rrp} = 2 - 3$)[18], synaptic noise remains the dominant source of noise driving the rapid chaotic divergence. In addition, $n_{\rm rrp}$ may vary between and across synapse types, but a systematic exploration thereof is beyond the scope of the present study.

Supplementary Figures

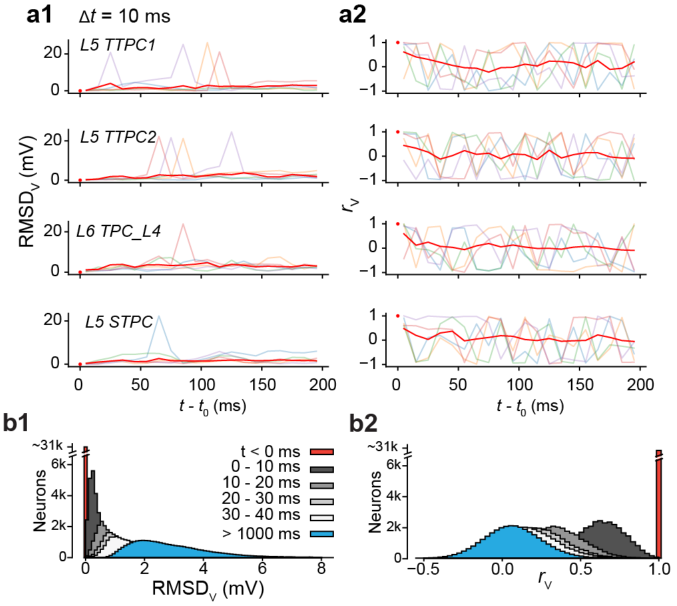

Supplementary Figure 1 – Rapid divergence of electrical activity. (**a**) Root-mean square deviation ($\mathrm{RMSD_V}$) and correlation (r_V) of the somatic membrane potentials between pairs of resumed simulations diverging from identical conditions, for five different base states (faded colors) and the mean of 40 saved base states (red), with $\Delta t = 10$ ms. Same neurons as in Fig. 1c. (**b**) Time evolution of distributions of mean $\mathrm{RMSD_V}$ and r_V values for individual neurons.

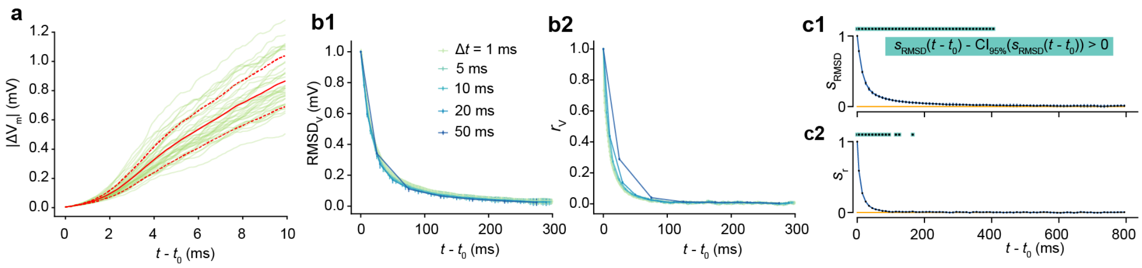

Supplementary Figure 2 – Quantifying the rapid divergence of electrical activity. (**a**) Mean divergence in the first 10 ms, with $\Delta t_\mathrm{V} = 0.1$ ms (mean of all neurons and 40 saved base states ± standard deviation). (**b**) $\mathrm{RMSD_V}$ and r_V for different analysis bin sizes Δt. The time step for the soma voltage is $\Delta t_\mathrm{V} = 0.1$ ms. (**c**) The similarity (s_RMSD and s_r) (mean ± 95% confidence interval). Dots signal time bins where s_RMSD and s_r are larger than 0, by a 95% confidence interval ($p < 0.025$; one-sided t-test).

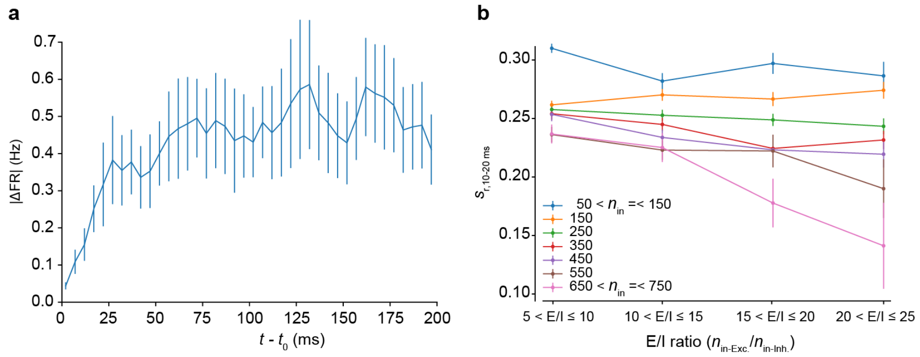

Supplementary Figure 3 – Rapid divergence of population activity; in-degree and EI-balance. (**a**) Mean population firing rate difference ($\Delta t = 5$ ms) between pairs of simulations diverging from identical initial conditions (mean of all neurons and of 40 saved base states ± 95% confidence interval). (**b**) Similarity s_r for subsets of neurons grouped by in-degree, and by the ratio of excitatory presynaptic connections over inhibitory connections (mean ± 95% confidence interval).

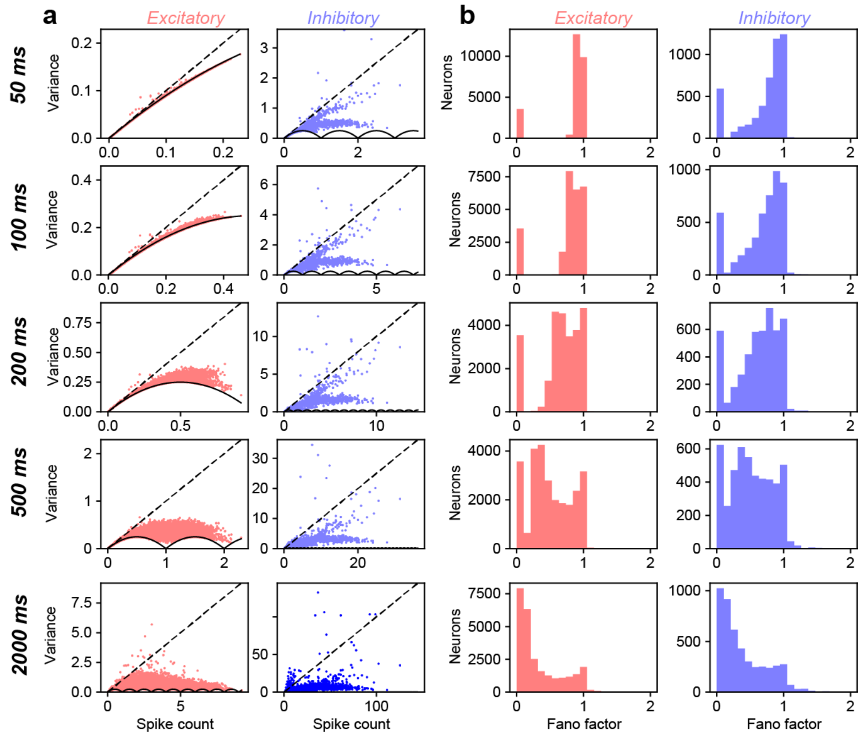

Supplementary Figure 4 – Fano factors. (**a**) Mean spike count and variance of spike count of 40 independent trials of increasing duration for all neurons in the microcircuit, plotted separately for excitatory neurons (red) and inhibitory neurons (blue). The dashed lines indicate the expected values for a Poisson process. (**b**) Distribution of Fano factors (variance divided by mean spike count) corresponding to **a**.

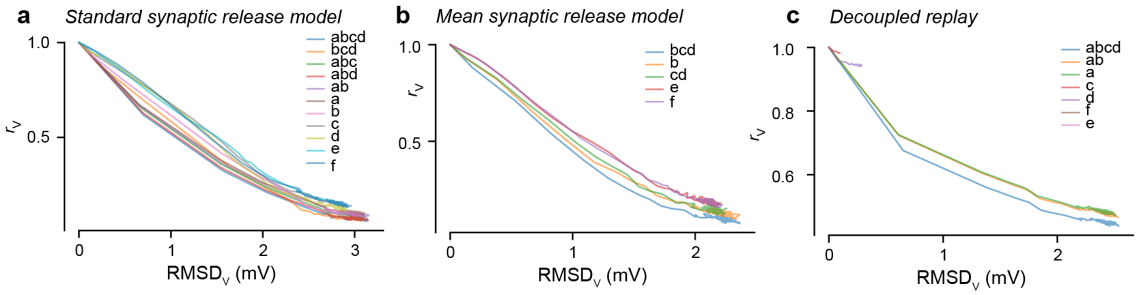

Supplementary Figure 5 – Linear relationship between $\mathbf{RMSD_V}$ and $r_\mathbf{V}$. Root-mean square deviation ($\mathrm{RMSD_V}$) and correlation (r_V) of the somatic membrane potentials between pairs of simulations diverging from identical initial conditions (mean of all neurons and saved base states). (**a**) Changing random seeds for subsets of noise sources with the standard stochastic release model. (**b**) Changing random seeds for subsets of noise sources with a mean release model. (**c**) Standard stochastic release model for decoupled, replayed simulations. *abcd*: 40 base states; all others: 20 base states.

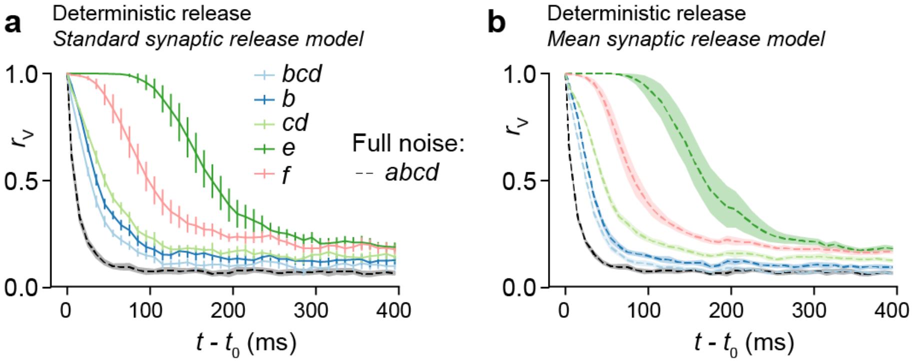

Supplementary Figure 6 – Mean synaptic release model. (**a**) Correlation r_V (as in Fig. 4 and Supplementary Fig. 5), with pseudo-deterministic synaptic release by not changing the random seeds for vesicle release (but with a change in '*mini*' signals for *b*). (**b**) As in **a**, but with deterministic synaptic release (mean release model), apart from *abcd* which has the fully stochastic model. Based on 20 saved base states (*abcd*: 40 saved base states); mean of base states $\pm$ 95% confidence interval.

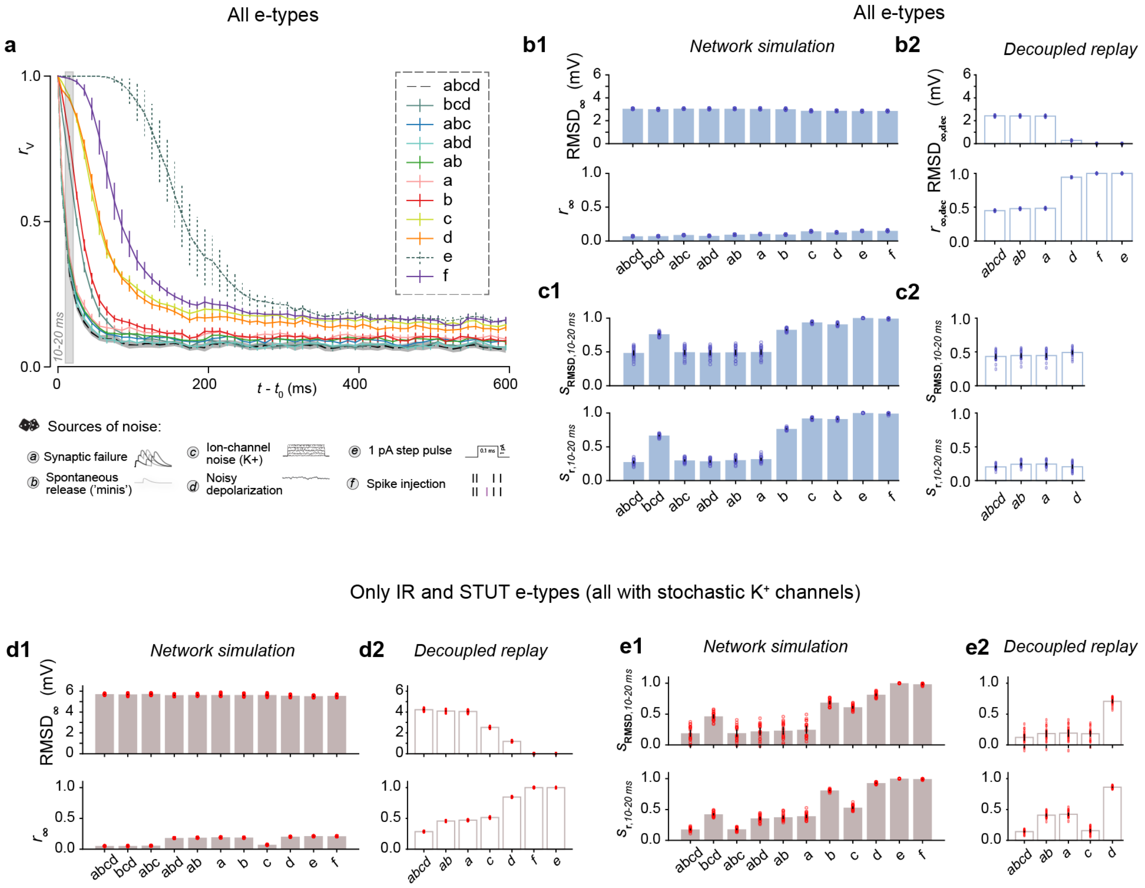

Supplementary Figure 7 – Unravelling noise sources. (**a**) Correlation r_V from identical initial conditions with different cellular noise sources turned on, and when turning of cellular noise, but perturbing the system by a single extra spike (in one neuron) or a miniscule perturbation in all neurons. (**b**) Steady-state membrane potential fluctuations (RMSD_∞) and correlations (r_∞) for network simulations (**b1**) and decoupled, replayed simulations (**b2**) for different noise sources. (**c**) Similarity $s_{\mathrm{r/RMSD}}$ at 10-20 ms for network simulations (**c1**) and decoupled, replayed simulations (**c2**) for different noise sources. (**d-e**) Same as **b**–**c**, but only for the subset of neurons that have stochastic ion-channels (irregularly firing e-types, 1'137 out of 31'346 neurons). All error bars indicate 95% confidence intervals, based on 20 pairs of simulations (40 for *abcd*). Blue and red dots indicate individual simulation pairs.

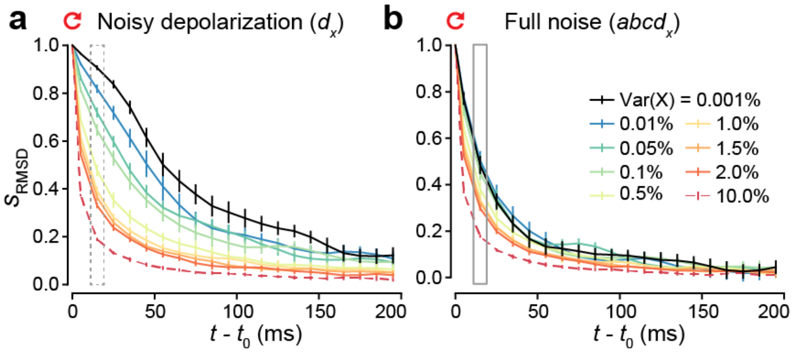

Supplementary Figure 8 – Predicting impact of other noise sources. (**a**) Similarity s_{RMSD} when only changing random seeds for noisy depolarization, but with different magnitudes of noise. (**b**) As in **a**, but with all noise sources enabled by changing random seeds. Based on 10 saved base states; mean of base states ± 95% confidence interval.

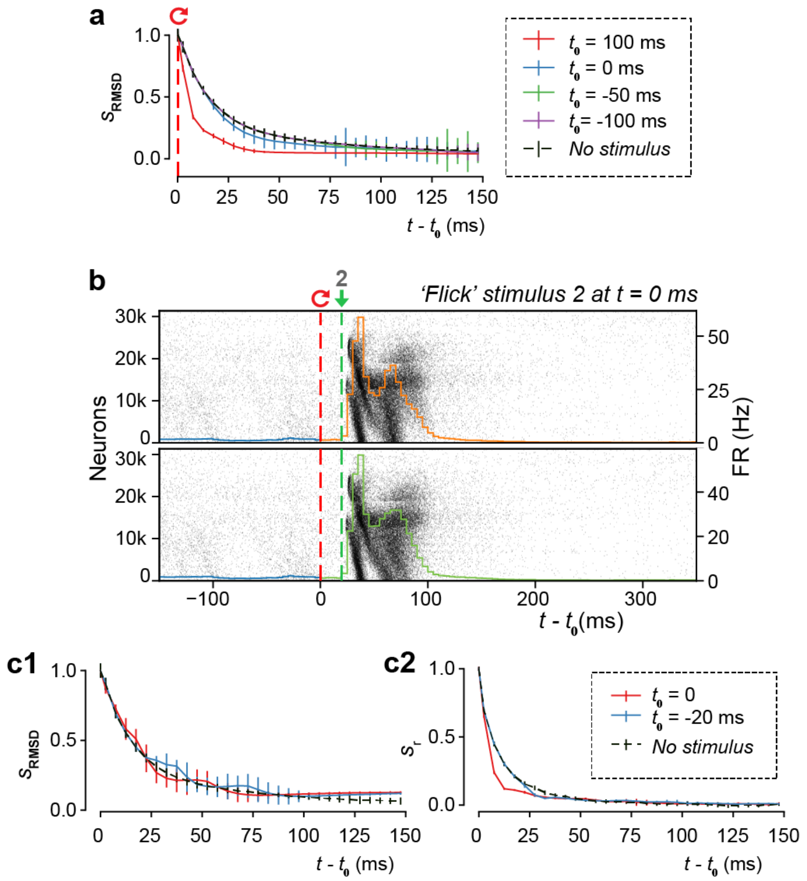

Supplementary Figure 9 – Divergence of evoked activity. (**a**) The similarity s_{RMSD} defined as the difference between the RMSD_{V} of diverging and independent trials, normalized to lie between 1 (identical) and 0 (fully diverged) (mean ± 95% confidence interval, 20 saved base states), for the thalamic stimulus. (**b**) Population raster plot and population peristimulus time histogram (PSTH) of all 31'346 neurons in the microcircuit, during evoked activity with a simplified "whisker flick" stimulus (60 VPM neurons are firing at the same time, one spike). (**c1**) As **a**, but for the "whisker flick" stimulus. (**c2**) As **c1**, but for s_r instead of s_{RMSD}.

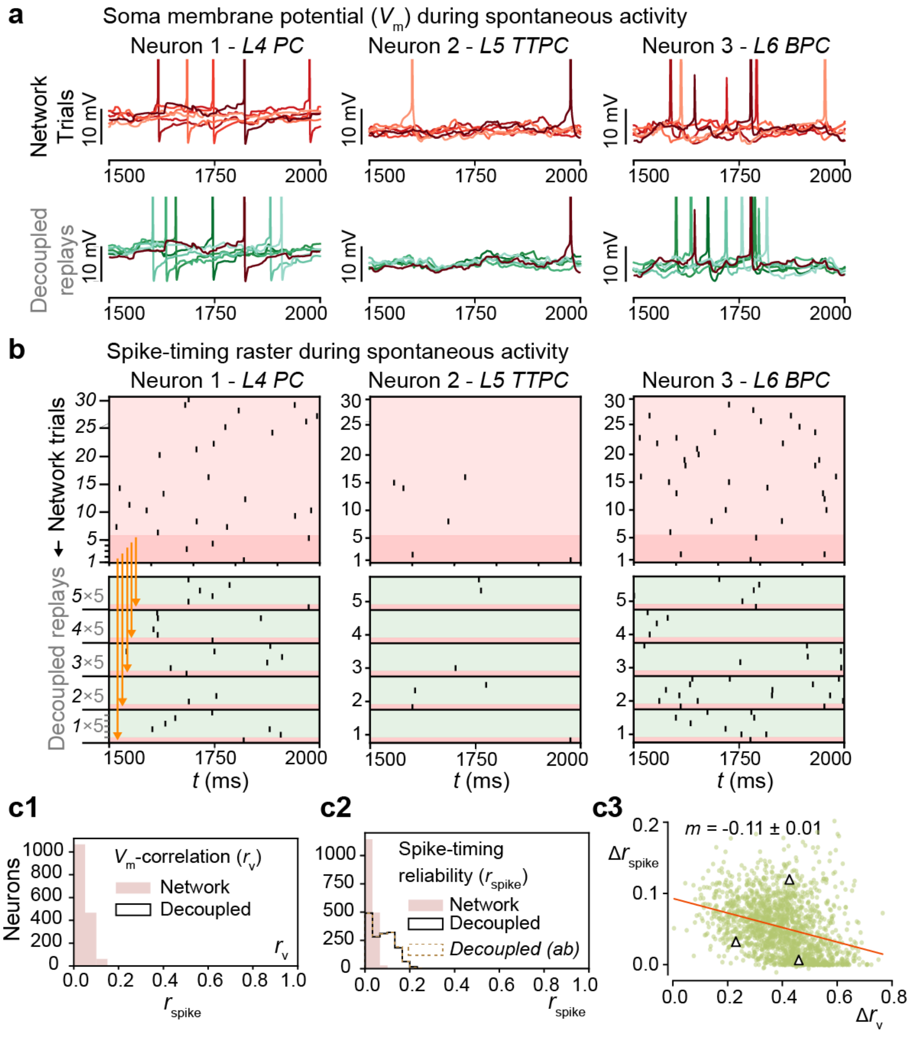

Supplementary Figure 10 – Low trial-by-trial spike-timing reliability during spontaneous activity. (**a**) Somatic membrane potentials (V_m) of three representative neurons. Top: during six independent trials of spontaneous activity. Bottom: five decoupled replay trials (green) with the same presynaptic input as during the original network simulation trial (red), but with different random seeds. (**b**) Top: Raster plot of spike times for the same example neurons as in **a**, during 30 independent trials of spontaneous activity. Bottom: 5 decoupled replay trials (green) of the same input received during 5 of the 30 original trials (dark red). (**c1**) Mean somatic membrane potential correlation r_V of the 1666 (*ab*: 1670) most central (and spiking) pyramidal neurons from layers 4, 5, and 6 between independent network simulations, and between decoupled replay simulations with identical presynaptic inputs. (**c2**) Mean spike-timing reliability r_{spike} of the same neurons. Decoupled and decoupled (ab) are overlapping. (**c3**) Change in correlation, Δr_V, versus change in spike-timing reliability, Δr_{spike}, for each neuron for decoupled replay simulations relative to network simulations (linear fit with 68% confidence interval on slope m, red line). Triangles indicate values of representative neurons in panel B.

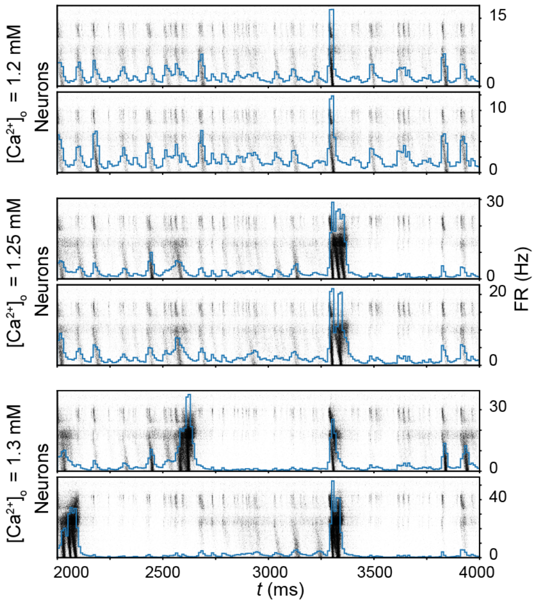

Supplementary Figure 11 – Population response at different calcium concentrations. Spike raster of all neurons in the microcircuit and mean population firing rate for two trials of evoked activity with the same thalamic stimulus, for three different extracellular calcium concentrations.

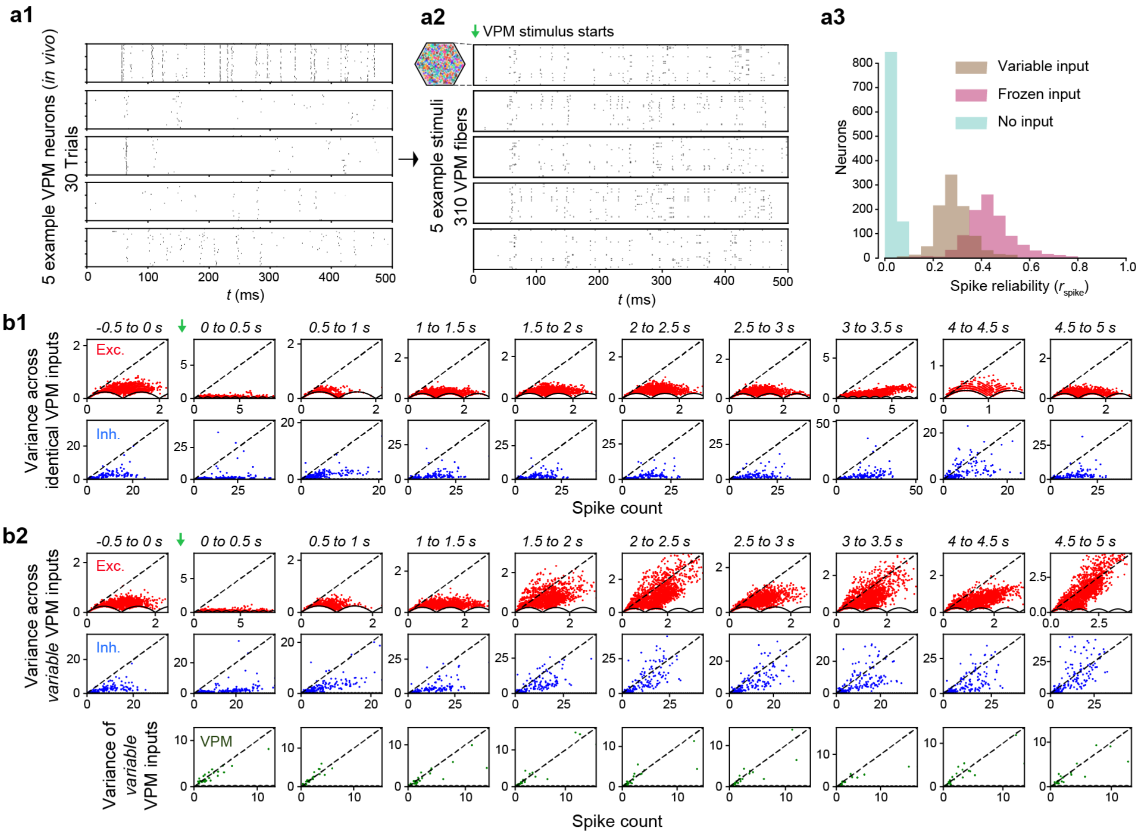

Supplementary Figure 12 – Variable thalamic input. (**a1**) In vivo recordings of five thalamic neurons during 30 trials of replayed whisker deflection. (**a2**) Variable input created from in vivo recordings. (**a3**) Spike-timing reliability for no input (spontaneous activity), frozen input (same as Figs. 5–8) and for variable input, for the same pyramidal neurons analyzed in Figs. 5–8. (**b1**) Variance vs. mean spike count of all neurons in the microcircuit over 30 trials with identical inputs (same input as in Figs. 5–8), for adjacent time bins of 500 ms duration. (**b2**) Variance vs. mean spike count with variable input, and Fano factors of VPM input, across 30 trials.

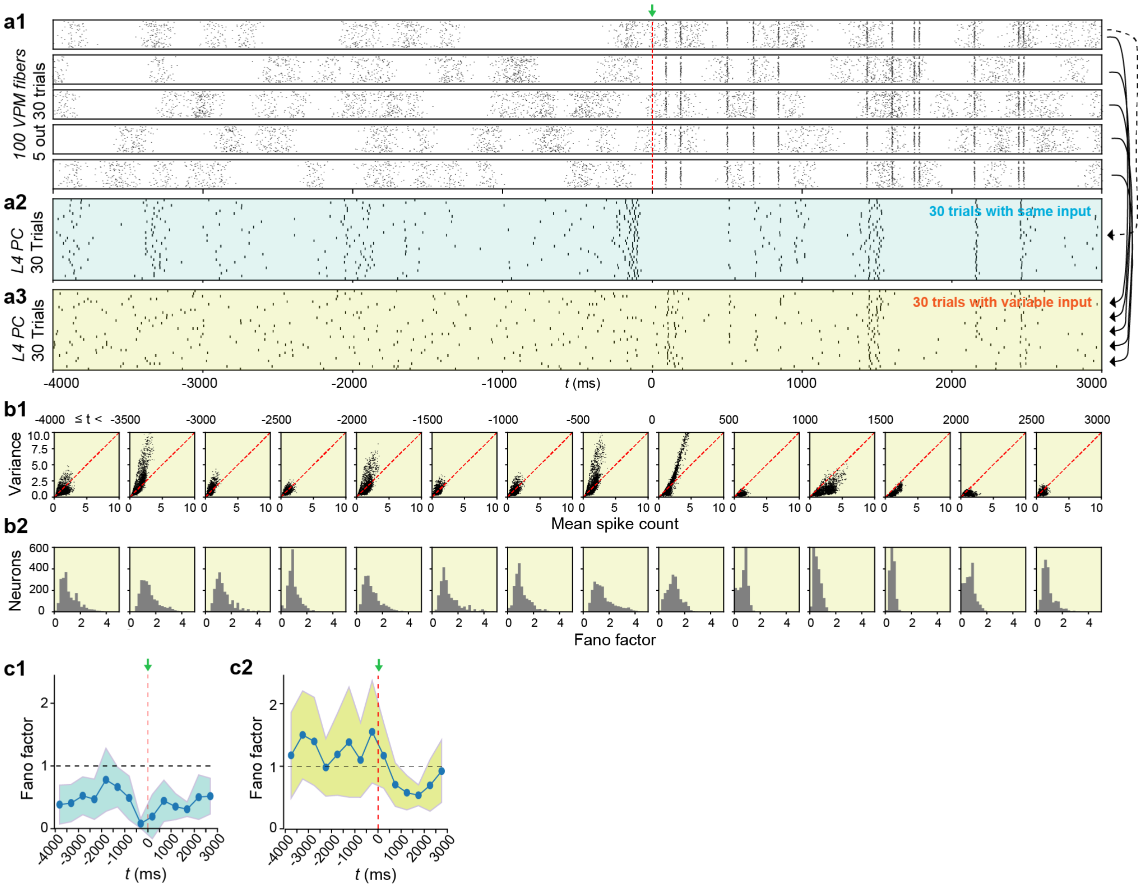

Supplementary Figure 13 – Quenching of spike count variability. (**a1**) The central 100 VPM fibers innervating the microcircuit are activated with different inputs: 60 randomly chosen fibers contain variable inputs that are correlated between the fibers (Poisson-spike train, FR = 4 Hz, $\sigma_{\text{corr}} = 50$ ms), and different in each of 30 trials (5 shown). The other 40 fibers contain a reliable signal (FR = 4 Hz) of randomly distributed spikes, that is highly correlated between the fibers ($\sigma_{\text{corr}} = 2$ ms) and always arriving at roughly the same time between trials. The variable input lasts from -4000 to 3000 ms, whereas the reliable input only starts after t = 0 ms. (**a2**) Spike response of example L4 PC across 30 trials to *one* of the 30 inputs from **a1** (top stimulus). (**a3**) Spike response of same example L4 PC across 30 trials to *all* 30 inputs from a1. (**b1**) Variance of spike count vs. mean spike count of central 2024 excitatory neurons in layers 4, 5, and 6 (as before) for the 30 variable input trials, split in bins of 500 ms duration. (**b2**) Fano factors for the same neurons and time bins as in **b1**. (**c1**) Mean Fano factor (blue) and standard deviation (light blue) across trials with the same identical input. (**c2**) Mean Fano factor (blue) and standard deviation (green) of the neurons in **b1** and **b2** across trials with variable input.

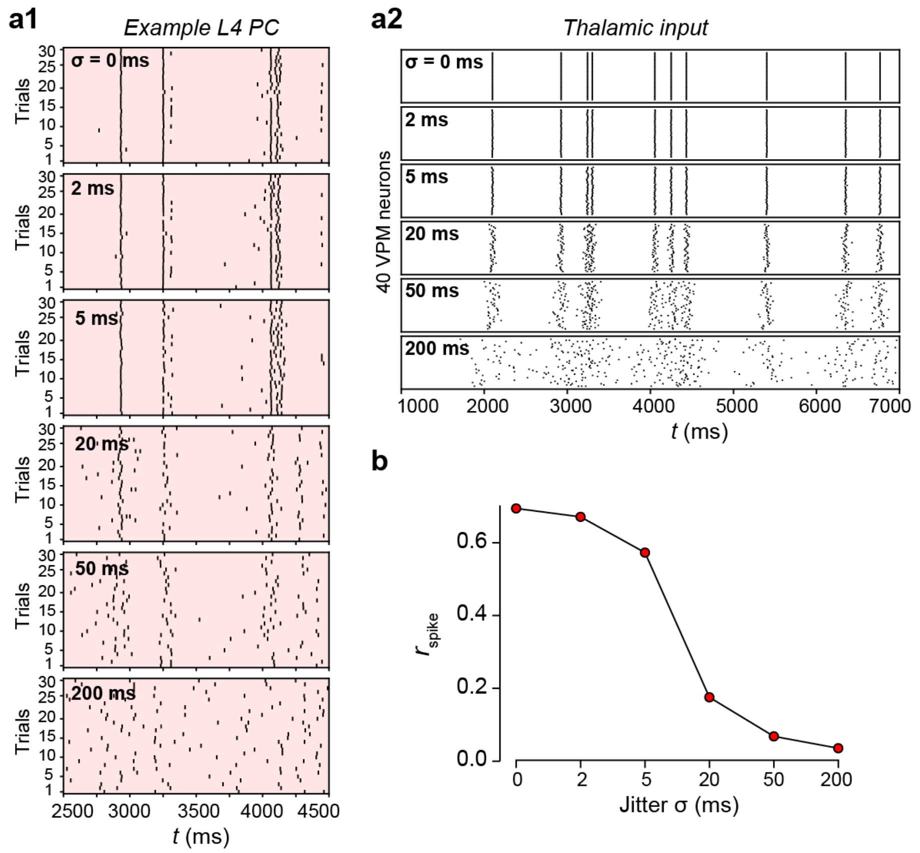

Supplementary Figure 14 – Input synchrony and reliability. (**a1**) Response of a layer 4 pyramidal cell to a simple thalamic stimulus consisting of 40 synchronous spikes with increasing jitter (but frozen across trials). (**a2**) The thalamic stimulus, with increasing normally-distributed jitter with standard deviation σ. The stimulus is kept identical across 30 repetitions to study only intrinsic cortical variability, as before. (**b**) Mean spike-timing reliability of 2024 pyramidal neurons from layers 4, 5 and 6 (as before) versus jitter standard deviation σ (mean of 30 trials, 95%-CI smaller than marker symbols).

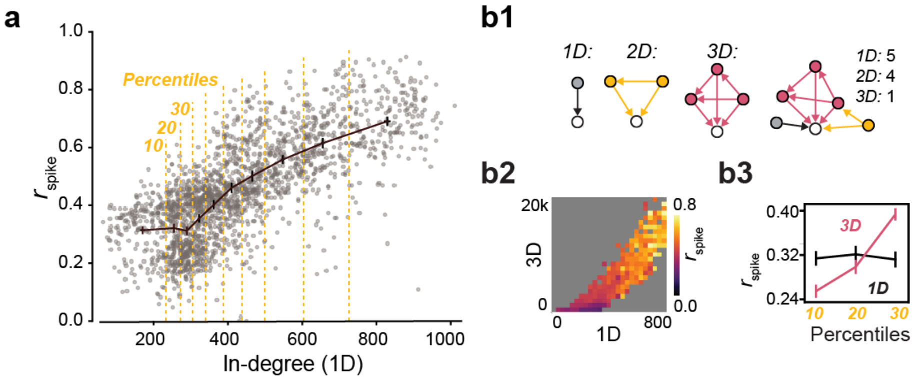

Supplementary Figure 15 – In-degree and higher-dimensional connectivity. (**a**) In-degree (number of presynaptic connections) vs. spike-timing reliability, for the same neurons as in Fig. 8. Mean of in-degree percentiles (the 10% of neurons with the lowest in-degree, the 10% of neurons with the second lowest in-degree, etc.). Error bars indicate 95%-confidence interval. (**b1**) Higher order connectivity. *1D*: number of presynaptic neurons (one-dimensional directed cliques). *2D*: number of two-dimensional directed cliques a neuron is the sink of. *3D*: Number of three-dimensional directed cliques a neuron is the sink of. Example network: the white neuron is the sink of five 1D-cliques, four 2D-cliques, and one 3D-cliques, according to the definition of directed cliques to the left. (**b2**) In-degree (1D) vs. number of 3D-directed-cliques a neuron is the sink of (3D). Raster plot shows mean reliability of all neurons in a pixel. Grey indicates that no neuron falls in this pixel. (**b3**) The 30% of neurons with the lowest indegree in black (same as first three percentiles in **a**). Red: The same neurons reordered according to number of 3D-directed-cliques a neuron is the sink of.

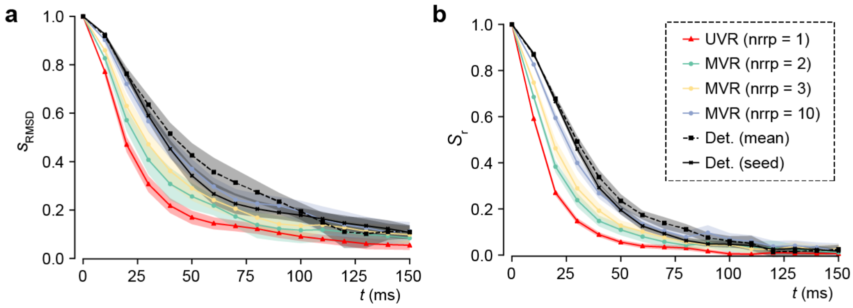

Supplementary Figure 16 – Multivesicular release. Change in divergence time course depending on the size of the pool of readily releasable vesicles (n_{rrp}), quantified by similarity of the somatic membrane potentials diverging from identical initial conditions: (**a**) s_{RMSD} and (**b**) s_{r}. (mean of all neurons and n base states $\pm$ 95% confidence interval; UVR: $n = 40$; all others: $n = 20$).

Supplementary References

1. Markram, H. *et al.* Reconstruction and Simulation of Neocortical Microcircuitry. *Cell* **163,** 456–492. ISSN: 00928674. `http://linkinghub.elsevier.com/retrieve/pii/S0092867415011915` (2016) (Oct. 2015).
2. Okun, M. *et al.* Diverse coupling of neurons to populations in sensory cortex. *Nature* **521,** 511–515. ISSN: 0028-0836. `http://www.nature.com/nature/journal/v521/n7553/abs/nature14273.html` (2016) (May 2015).
3. Mainen, Z. F. & Sejnowski, T. J. Reliability of spike timing in neocortical neurons. *Science* **268,** 1503–1506. ISSN: 0036-8075, 1095-9203. `http://www.sciencemag.org/content/268/5216/1503` (2014) (June 1995).
4. Churchland, M. M. *et al.* Stimulus onset quenches neural variability: a widespread cortical phenomenon. *Nature Neuroscience* **13,** 369–378. ISSN: 1097-6256. `http://www.nature.com/neuro/journal/v13/n3/abs/nn.2501.html` (2017) (Mar. 2010).
5. Musall, S., Kaufman, M. T., Gluf, S. & Churchland, A. K. Movement-related activity dominates cortex during sensory-guided decision making. *bioRxiv,* 308288. `https://www.biorxiv.org/content/early/2018/05/10/308288` (2018) (May 2018).
6. Bale, M. R. & Petersen, R. S. Transformation in the Neural Code for Whisker Deflection Direction Along the Lemniscal Pathway. *Journal of Neurophysiology* **102,** 2771–2780. ISSN: 0022-3077, 1522-1598. `http://jn.physiology.org/content/102/5/2771` (2015) (Nov. 2009).
7. Fairhall, A. L. Whither variability? *Nature Neuroscience* **22,** 329. ISSN: 1546-1726. `https://www.nature.com/articles/s41593-019-0344-0` (2019) (Mar. 2019).
8. Muller, L., Chavane, F., Reynolds, J. & Sejnowski, T. J. Cortical travelling waves: mechanisms and computational principles. *Nature Reviews Neuroscience* **19,** 255–268. ISSN: 1471-0048. `https://www.nature.com/articles/nrn.2018.20/` (2019) (May 2018).
9. Masquelier, T. Neural variability, or lack thereof. *Frontiers in Computational Neuroscience* **7.** ISSN: 1662-5188. `https://www.frontiersin.org/articles/10.3389/fncom.2013.00007/full` (2017) (2013).
10. Faisal, A. A., Selen, L. P. J. & Wolpert, D. M. Noise in the nervous system. *Nature Reviews Neuroscience* **9,** 292–303. ISSN: 1471-003X. `http://www.nature.com/nrn/journal/v9/n4/abs/nrn2258.html` (2015) (Apr. 2008).
11. Mendonça, P. R. *et al.* Stochastic and deterministic dynamics of intrinsically irregular firing in cortical inhibitory interneurons. *eLife* **5,** e16475. ISSN: 2050-084X. `https://elifesciences.org/articles/16475` (2017) (Aug. 2016).
12. Diba, K., Koch, C. & Segev, I. Spike propagation in dendrites with stochastic ion channels. *Journal of Computational Neuroscience* **20,** 77–84. ISSN: 0929-5313, 1573-6873. `https://link.springer.com/article/10.1007/s10870-006-4770-0` (2017) (Feb. 2006).
13. Cox, C. L., Denk, W., Tank, D. W. & Svoboda, K. Action potentials reliably invade axonal arbors of rat neocortical neurons. *Proceedings of the National Academy of Sciences* **97,** 9724–9728. ISSN: 0027-8424, 1091-6490. `http://www.pnas.org/content/97/17/9724` (2016) (Aug. 2000).
14. Faisal, A. A. & Laughlin, S. B. Stochastic Simulations on the Reliability of Action Potential Propagation in Thin Axons. *PLOS Computational Biology* **3,** e79. ISSN: 1553-7358. `http://journals.plos.org/ploscompbiol/article?id=10.1371/journal.pcbi.0030079` (2016) (May 2007).
15. Rudolph, S., Tsai, M.-C., von Gersdorff, H. & Wadiche, J. I. The ubiquitous nature of multivesicular release. *Trends in Neurosciences* **38,** 428–438. ISSN: 0166-2236. `http://www.sciencedirect.com/science/article/pii/S0166223615001228` (2018) (July 2015).

16. Silver, R. A., Lübke, J., Sakmann, B. & Feldmeyer, D. High-Probability Uniquantal Transmission at Excitatory Synapses in Barrel Cortex. *Science* **302,** 1981–1984. ISSN: 0036-8075, 1095-9203. http://science.sciencemag.org/content/302/5652/1981 (2018) (Dec. 2003).

17. Loebel, A. *et al.* Multiquantal release underlies the distribution of synaptic efficacies in the neocortex. *Frontiers in Computational Neuroscience* **3.** ISSN: 1662-5188. https://www.frontiersin.org/articles/10.3389/neuro.10.027.2009/full (2018) (2009).

18. Barros-Zulaica, N. *et al.* Estimating the Readily-Releasable Vesicle Pool Size at Synaptic Connections in a Neocortical Microcircuit. *bioRxiv,* 646497. https://www.biorxiv.org/content/10.1101/646497v1 (2019) (May 2019).
